# Supplementary figures and images for: SIRT2, ERK and Nrf2 Mediate NAD+ Treatment-Induced Increase in the Antioxidant Capacity of PC12 Cells Under Basal Conditions
Source: Front Mol Neurosci. 2019 Apr 26;12:108. doi: 10.3389/fnmol.2019.00108 (PMC6497790; doi:10.3389/fnmol.2019.00108)

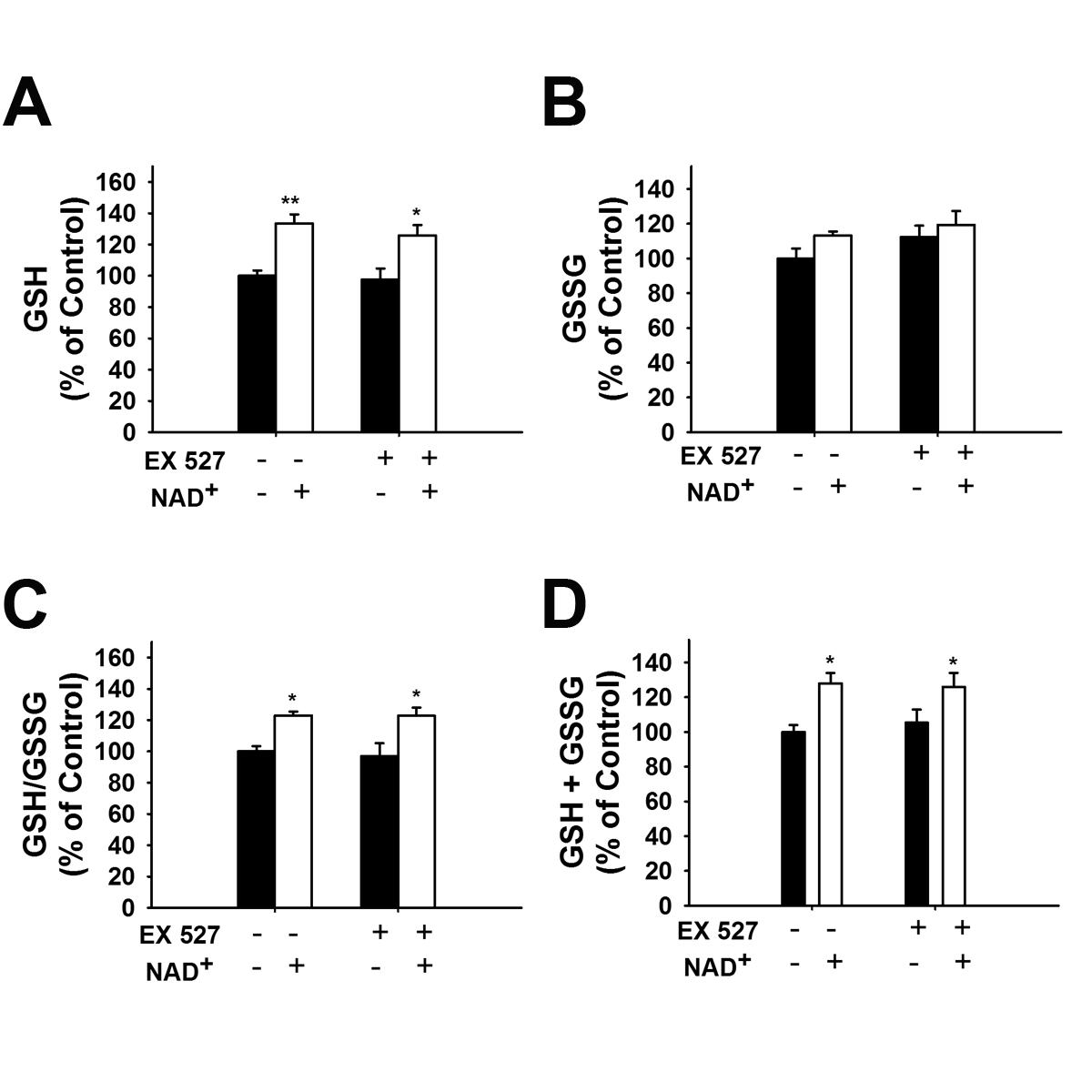

Supplement: FIGURE S1 — EX 527, a SIRT1 inhibitor, did not prevent the NAD+-induced increases in the GSH level, total glutathione level, and GSH/GSSG ratio in PC12 cells. (A) EX 527 did not prevent the NAD+-induced increase in the GSH level of the cells (effect of NAD+ treatment, F(1,32) = 27.53, p < 0.0001; effect of EX527 treatment, F(1,32) = 0.7361, p = 0.3973; effect of EX527 * NAD+ treatment, F(1,32) = 0.2272, p = 0.6369). (B) Effects of EX 527 and NAD+ on GSSG levels (effect of NAD+ treatment, F(1,32) = 2.853, p = 0.1009; effect of EX527 treatment, F(1,32) = 2.364, p = 0.1340; effect of EX527 * NAD+ treatment, F(1,32) = 0.2706, p = 0.6065). (C) EX 527 did not prevent the NAD+-induced increase in the GSH/GSSG ratio of the cells (effect of NAD+ treatment, F(1,32) = 21.13, p < 0.0001; effect of EX527 treatment, F(1,32) = 0.09369, p = 0.7615; effect of EX527 * NAD+ treatment, F(1,32) = 0.07047, p = 0.7924). (D) EX 527 did not prevent the NAD+-induced increase in the total glutathione level of the cells (effect of NAD+ treatment, F(1,32) = 13.66, p = 0.0008; effect of EX527 treatment, F(1,32) = 0.06223, p = 0.8046; effect of EX527 * NAD+ treatment, F(1,32) = 0.3151, p = 0.5785). The assays were conducted after the cells were treated with 1 mM NAD+ and 5 μM EX 527 for 24 h. The data were pooled from four independent experiments. *p < 0.05; **p < 0.01. [file Image_1.TIF]

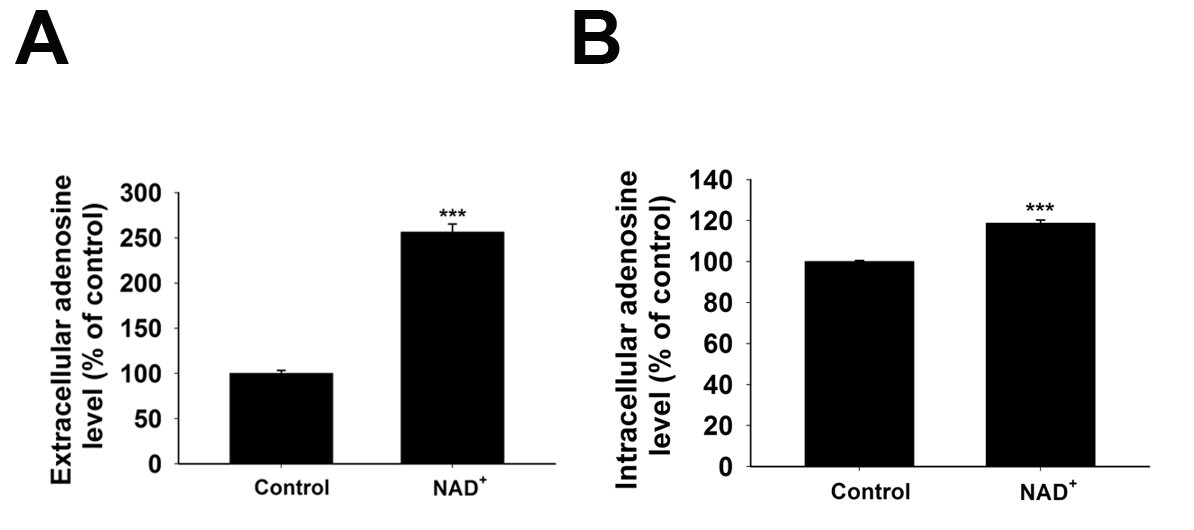

Supplement: FIGURE S2 — NAD+ treatment led to a significant increase in the intracellular and extracellular adenosine levels in PC12 cells. After the cells were treated with 1 mM NAD+ for 24 h, the intracellular and extracellular adenosine levels were determined. (A) NAD+ treatment significantly increased the extracellular adenosine level in PC12 cells. (B) NAD+ treatment significantly increased the extracellular adenosine level in PC12 cells. The data were pooled from four independent experiments. ***p < 0.001. [file Image_2.TIF]

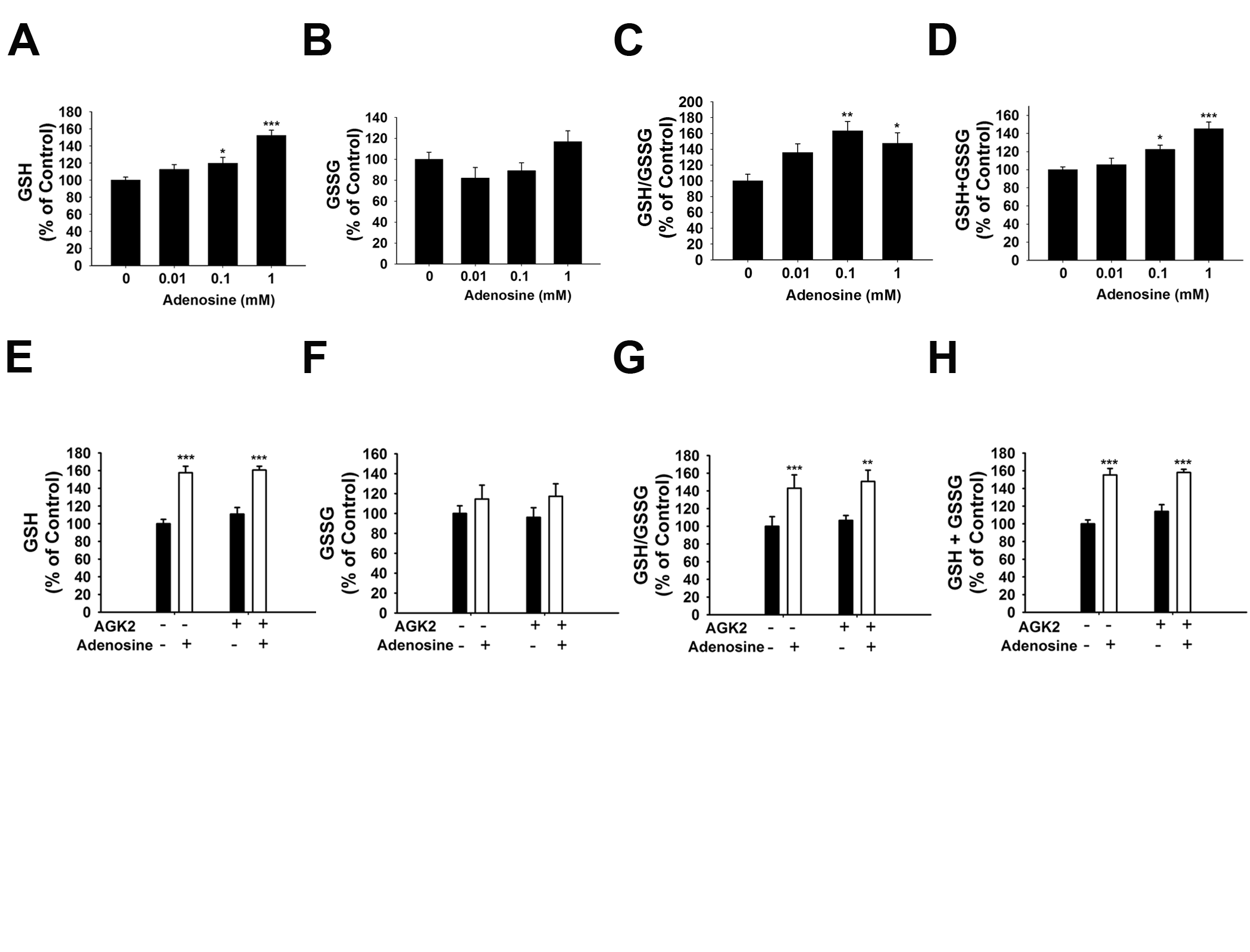

Supplement: FIGURE S3 — AGK2 could not block the adenosine-produced increases in the glutathione level and GSH/GSSG ratio. (A) Adenosine treatment increased the GSH levels in the cells. (ANOVA: F = 15.33, p < 0.0001). (B) Adenosine did not affect the GSSG levels (ANOVA: F = 2.902, p = 0.0499). (C) Adenosine significantly increased the GSH/GSSG ratio in the cells (ANOVA: F = 5.663, p = 0.0031). (D) Adenosine treatment dose-dependently increased the total glutathione level of the cells (ANOVA: F = 11.8, p < 0.0001). (E) AGK2 did not prevent the adenosine-induced increase in the GSH level of the cells (effect of adenosine treatment, F(1,32) = 75.71, p < 0.0001; effect of AGK2 treatment, F(1,32) = 1.24, p = 0.2738; effect of AGK2 * adenosine treatment, F(1,32) = 0.4104, p = 0.5263). (F) The GSSG level was not affected by AGK2 or adenosine (effect of adenosine treatment, F(1,32) = 2.528, p = 0.1217; effect of AGK2 treatment, F(1,32) = 0.002108, p = 0.9637; effect of AGK2 * adenosine treatment, F(1,32) = 0.08424, p = 0.7735). (G) AGK2 did not prevent the adenosine-induced increase in the GSH/GSSG ratio of the cells (effect of adenosine treatment, F(1,32) = 23.93, p < 0.0001; effect of AGK2 treatment, F(1,32) = 0.6463, p = 0.4274; effect of AGK2* adenosine treatment, F(1,32) = 0.00351, p = 0.9531). (H) AGK2 did not prevent the adenosine-induced increase in the total glutathione level of the cells (effect of adenosine treatment, F(1,32) = 69.45, p < 0.0001; effect of AGK2 treatment, F(1,32) = 1.994, p = 0.1676; effect of AGK2 * adenosine treatment, F(1,32) = 0.9088, p = 0.3476). The cells were treated with 1 mM adenosine with or without 5 μM AGK2 for 24 h. The data were pooled from four independent experiments. *p < 0.05; **p < 0.01; ***p < 0.001. [file Image_3.TIF]

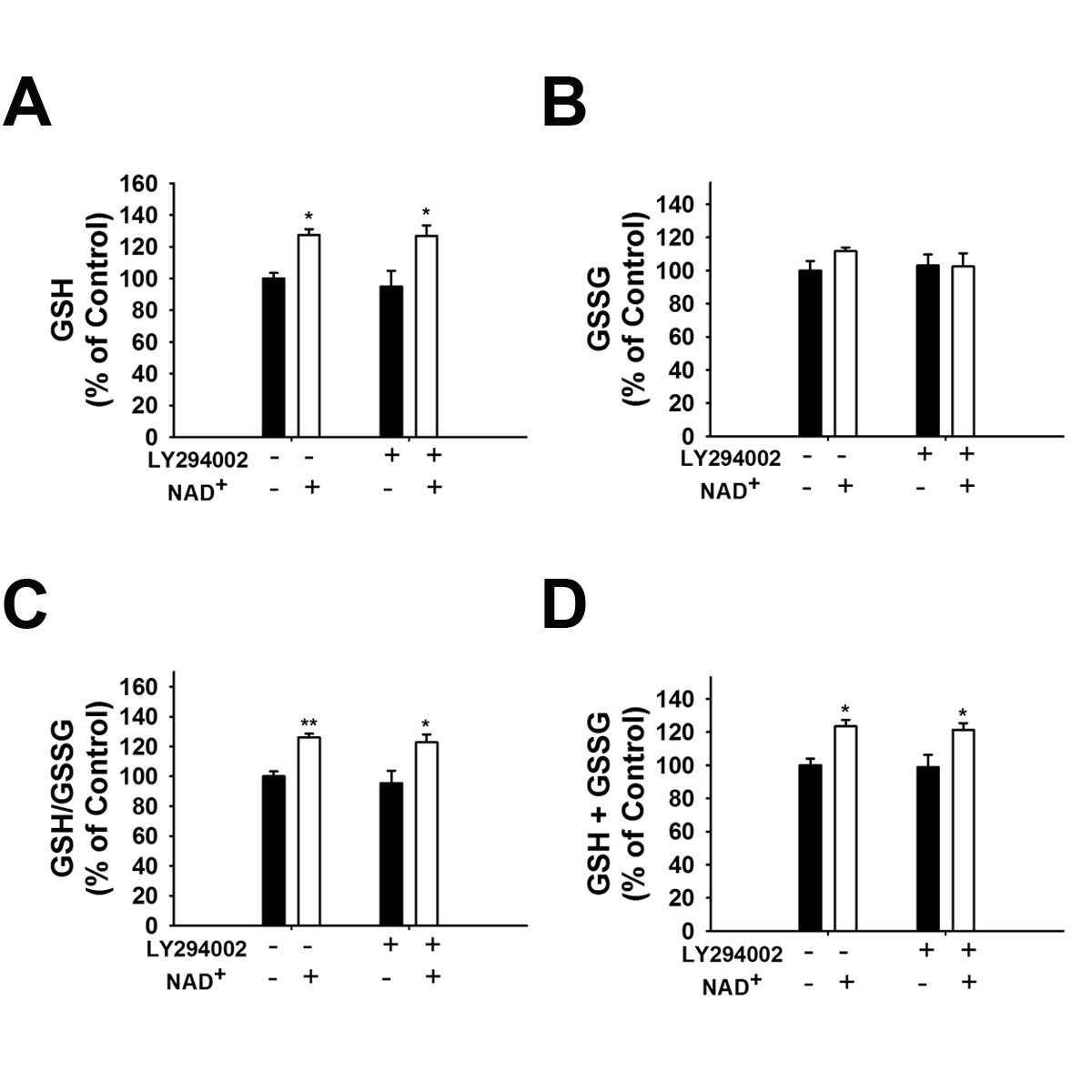

Supplement: FIGURE S4 — LY294002, a PI3K/Akt pathway inhibitor, did not prevent the NAD+-induced increases in the glutathione levels and GSH/GSSG ratio in PC12 cells. (A) LY294002 did not prevent the NAD+-induced increase in the GSH level of the cells (effect of NAD+ treatment, F(1,32) = 20.74, p < 0.0001; effect of LY294002 treatment, F(1,32) = 0.1793, p = 0.6748; effect of LY294002 * NAD+ treatment, F(1,32) = 0.1216, p = 0.7296). (B) Effects of LY294002 and NAD+ on GSSG levels (effect of NAD+ treatment, F(1,32) = 0.8547, p = 0.3621; effect of LY294002 treatment, F(1,32) = 0.2553, p = 0.6168; effect of LY294002* NAD+ treatment, F(1,32) = 1.035, p = 0.3167). (C) LY294002 did not prevent the NAD+-induced increase in the GSH/GSSG ratio of the cells (effect of NAD+ treatment, F(1,32) = 25.27, p < 0.0001; effect of LY294002 treatment, F(1,32) = 0.5421, p = 0.4669; effect of LY294002 * NAD+ treatment, F(1,32) = 0.01279, p = 0.9107). (D) LY294002 did not prevent the NAD+-induced increase in the total glutathione level of the cells (effect of NAD+ treatment, F(1,32) = 20.24, p < 0.0001; effect of LY294002 treatment, F(1,32) = 0.1071, p = 0.7456; effect of LY294002 * NAD+ treatment, F(1,32) = 0.008013, p = 0.9292). The assays were conducted, after the cells were treated with 1 μM LY294002 and 1 mM NAD+ for 24 h. The data were pooled from four independent experiments. *p < 0.05; **p < 0.01. [file Image_4.TIF]
